# Characterization and Factors Associated with Poor Asthma Control in Adults with Severe Eosinophilic Asthma

**DOI:** 10.3390/jpm13071173

**Published:** 2023-07-22

**Authors:** Clara Padró-Casas, María Basagaña, María Luisa Rivera-Ortún, Ignasi García-Olivé, Carlos Pollan-Guisasola, Aina Teniente-Serra, Eva Martínez-Cáceres, José-Tomás Navarro, Jorge Abad-Capa, Antoni Rosell, Albert Roger, Carlos Martínez-Rivera

**Affiliations:** 1 Severe Asthma Unit, Allergy Section, Hospital Universitari Germans Trias i Pujol, The Germans Trias i Pujol Research Institute (IGTP), Carretera de Canyet s/n, E-08916 Badalona, Spain; mbasagana.germanstrias@gencat.cat (M.B.); aroger.germanstrias@gencat.cat (A.R.); 2 Severe Asthma Unit, Department of Pneumology, Hospital Universitari Germans Trias i Pujol, The Germans Trias i Pujol Research Institute (IGTP), Universitat Autònoma de Barcelona, Carretera de Canyet s/n, E-08916 Badalona, Spainigarcia.germanstrias@gencat.cat (I.G.-O.); jabadc.germanstrias@gencat.cat (J.A.-C.); arosellg.germanstrias@gencat.cat (A.R.); cmartinez.germanstrias@gencat.cat (C.M.-R.); 3 Severe Asthma Unit, Department of Otorhinolaryngology, Hospital Universitari Germans Trias i Pujol, Carretera de Canyet s/n, E-08916 Badalona, Spain; cpollan.guisasola@gencat.cat; 4 Severe Asthma Unit, Immunology Department, Hospital Universitari Germans Trias i Pujol, The Germans Trias i Pujol Research Institute (IGTP), Carretera de Canyet s/n, E-08916 Badalona, Spain; ateniente.germanstrias@gencat.cat; 5 Severe Asthma Unit, Head of the Immunology Department, Department of Cell Biology, Physiology and Immunology, Hospital Universitari Germans Trias i Pujol, The Germans Trias i Pujol Research Institute (IGTP), Associate Professor of Immunology, Universitat Autònoma de Barcelona, Carretera de Canyet s/n, E-08916 Badalona, Spain; emmartinez.germanstrias@gencat.cat; 6 Department of Hematology, Institut Català d’Oncologia, Hospital Universitari Germans Trias i Pujol, Universitat Autònoma de Barcelona, Carretera de Canyet s/n, E-08916 Badalona, Spain; jnavarro@iconcologia.net

**Keywords:** eosinophilic asthma, biomarkers, blood eosinophils, fractional exhaled nitric oxide, asthma control, periostin

## Abstract

A study was conducted in 98 adult patients diagnosed with severe eosinophilic asthma (73.5% women, mean age 47.2 years) and followed prospectively for 1 year. The aim of the study was to characterize this population and to identify factors associated with poor prognosis at 1 year of follow-up. At the initial visit, uncontrolled severe asthma was diagnosed in 87.7% of patients. Allergic sensitization was observed in 81.7% (polysensitization in 17.3%), with clinically significant allergic asthma in 45%. The mean percentage of sputum eosinophils was 4.7% (standard deviation(SD) 6.3%) and the mean (SD) blood eosinophil count 467 (225) cells/µL. Almost half of the patients (48.3%) had sputum eosinophilia (>3% eosinophils). Sputum eosinophils correlated significantly with peripheral eosinophilia (*p* = 0.004) and, to a lesser extent, with fractional exhaled nitric oxide (FeNO) (*p* = 0.04). After 1 year, 48 patients (49%) had uncontrolled asthma in all visits, and 50 (51%) had controlled asthma in some visits. Airway obstruction (FEV_1_ < 80% predicted) was the main reason for uncontrolled asthma. In the multivariate analysis, an obstructive pattern (odds ratio (OR) 7.45, 95% confidence interval (CI) 2.41–23.03, *p* < 0.0001) and the patient’s age (OR 1.045, 95% CI 1.005–1.086, *p* = 0.026) were independent predictors of poor asthma control. In adult-onset and long-standing asthma, serum interleukin (IL) IL-17 was higher in the uncontrolled asthma group. This study contributes to characterizing patients with severe eosinophilic asthma in real-world clinical practice.

## 1. Introduction

Asthma encompasses distinct pathological conditions with different pathophysiological mechanisms (endotypes) expressing different clinical characteristics (phenotypes) [1,2,3]. Severe asthma is classified into an independent category in which the symptoms are not controlled with the use of corticosteroids, a pillar drug in the treatment of asthma. The prevalence of severe asthma among all patients with current asthma is about 7.7% [4]. Severe asthma is associated with high economic burden and remains a major public health concern [5]. Treatment of asthma—especially in patients with mild and moderate disease, but also in severe asthma—is based on a stepwise approach. In recent years, specific treatments targeted at patient endotypes have been introduced for the management of severe uncontrolled asthma [1,3]. The recognition of biomarkers to identify endotypes that could guide treatment has been a clinical research priority in the last years [6].

Almost two decades ago, Wenzel et al. [7] stratified corticosteroid-dependent asthma into two different subtypes based on the presence of eosinophilia in the respiratory tract. It has been recently shown that innate immunity, and not only adaptive immunity, also has a prominent role in the pathophysiology of asthma, with complex interconnections between them. T helper 2 (Th2) cells and type 2 innate lymphoid cells (ILC2) are the major regulators of type 2 immunity. T2 inflammation characterized by eosinophilic airway infiltrate is seen in 37% of patients with severe asthma [8]. Currently available biomarkers provide a platform for the characterization of T2-high and T2-low asthma phenotypes.

Some of these biomarkers have shown a prognostic value to assess the response to treatment and the evolution of the disease. For example, the demonstration of sputum eosinophils is considered the gold standard for the diagnosis of type 2 asthma [9]. Peripheral eosinophilia, blood level of periostin, fractional concentration of exhaled nitric oxide (FeNO), and allergen-specific IgE levels have been used as surrogates for sputum eosinophils in asthma [10]. However, prognostic and predictive information of these biomarkers is not fully interchangeable as they may be affected by individual clinical characteristics [11,12].

Sputum induction with hypertonic saline is a useful and non-invasive method to assess airway eosinophilia [13], but it is time consuming and requires trained staff, and some patients are unable to spontaneously expectorate adequate sputum samples.

The present prospective study was designed to add evidence of the clinical characterization of adult patients with severe eosinophilic asthma; to assess the correlation between sputum eosinophils and FeNO, peripheral eosinophilia, and serum periostin; and to identify prognostic biomarkers of asthma control.

## 2. Materials and Methods

### 2.1. Design and Participants

This was a single-center prospective cohort study of adult patients with severe asthma attended at the severe asthma unit of an acute tertiary care hospital in Badalona (Barcelona, Spain) and was followed over a 12-month period. Eligibility criteria were 18 years of age or older, diagnosis of severe asthma according to guidelines of the Global Initiative for Asthma (GINA) [1] established at least 1 year before inclusion in the study, peripheral eosinophilia (≥220 cells/µL) [10], and agreement to sign the informed consent. Patients attended the specialized severe asthma unit and underwent a nursing visit in parallel with the specialist visit, in which the test of adherence to inhalers (TAI) [14] was completed (to assess medication compliance), and received education on inhaler technique. Moreover, all patients were required to have their treatment optimized at the time of selection. Asthma that remains uncontrolled despite optimized treatment with high-dose ICS-LABA or that requires high-dose ICS-LABA to prevent it from becoming uncontrolled was defined as severe asthma [1].

The study was conducted in accordance with the Declaration of Helsinki and was approved by the Ethics Committee for Clinical Research (CEIC) of the Hospital Universitari Germans Trias i Pujol (Badalona, Spain) (code PI-15-076, approval 10 July 2015). Written informed consent was obtained from all participants.

### 2.2. Study Procedures

The study included an initial visit and three visits scheduled every 4 months over the 1-year follow-up period. At each visit, the following were recorded: personalized medical history; the Asthma Control Test (ACT) [15] (an ACT score ≥ 20 indicates well-controlled asthma) and the Asthma Quality of Life Questionnaire (AQLQ) [16] (scores range 1–7 with higher scores indicating better quality of life) using validated Spanish versions of both instruments; physical examination, including assessment of body mass index (BMI); pulmonary function tests; and measurement of FeNO following guidelines of the European Respiratory Society and the American Thoracic Society (ERS/ATS) [2]. Additionally, asthma control was evaluated at each visit according to the International ERS/ATS guidelines [2]. Uncontrolled asthma was defined in the presence of at least one of the following characteristics: (1) poor control of symptoms with an ACT score < 20; (2) frequent severe exacerbations requiring two or more courses of systemic corticoids for more than 3 days since the last visit; (3) severe exacerbations requiring admission to the hospital or an intensive care unit (ICU) or mechanical ventilation since the last visit; and (4) airflow limitation with forced expiratory volume in one second (FEV_1_) less than 80% of predicted (in the presence of FEV_1_/forced vital capacity (FVC) below the normal value) after bronchodilation with a short-acting beta agonist. Controlled asthma was defined in the presence of an ACT score between 20 and 25, absence of severe exacerbations, and absence of airflow limitation on pulmonary function testing.

All patients underwent an immunoallergic study using the pick test with a standard battery of allergens [17]. Results of the immunoallergic study in sensitized patients were evaluated by two experienced independent allergologists who also conducted a personalized interview to identify clinically relevant sensitizations. Measurement of serum levels of total IgE and specific IgE when patients presented positivity to more than three allergens or allergen component testing (ISAC assay, Thermo Fisher Scientific, Waltham, MA, USA) in polysensitized patients (≥3 allergen families).

Patients were also evaluated by an otorhinolaryngologist to assess the presence of nasal polyps or other significant anatomic abnormalities.

For induced sputum analysis, samples were obtained via inhalation of increasing concentrations of hypertonic saline (3%, 4%, 5%) based on the technique described by Pizzichini et al. [18] and Djukanović et al. [19] and adapted according to the manual of procedures of the Spanish Society of Pneumology and Thoracic Surgery (SEPAR) [20]. The sputum cellularity (%) was determined via staining with May–Grünwald–Giemsa and microscopic quantification. The criterion of induced sputum viability was established as <15% epithelial cells. Patients were divided into four groups according to sputum cellularity, which included a neutrophilic profile (eosinophils < 3%/neutrophils > 61%), an eosinophilic profile (eosinophils > 3%/neutrophils < 61%), a mixed granulocytic profile (eosinophils > 3%/neutrophils > 61%), and a pauci-granulocytic profile (eosinophils < 3%/neutrophils < 61%). Additionally, interleukins (IL) IL-4, IL-5, IL-8, IL-9, IL-13, and IL-17F were measured in induced sputum using the cytometric bead array (CBA) system, with results expressed as pg/mL. The criterion for induced sputum sample viability for analysis of IL was <40% cell death.

On the same day of sputum analysis, fasting peripheral blood samples were obtained for complete blood cell count, serum levels of periostin using a Periostin Human ELISA kit (Thermo Fisher Scientific), and serum IL-4, IL-5, IL-8, IL-9, IL-13, and IL-17 were measured with the CBA system. Blood samples were also obtained for the identification of cell populations (Th1, Th2, Th17, ILC1, ILC2, and ILC3) in fresh peripheral whole blood via flow cytometry.

### 2.3. Study Outcomes

The outcomes of the study were as follows: (a) to describe the clinical characteristics of patients with severe eosinophilic asthma, and (b) to identify predictors of poor asthma control after 12 months of follow-up.

### 2.4. Statistical Analysis

Categorical variables are expressed as frequencies and percentages, and continuous variables were expressed as mean and standard deviation (SD). The Student’s *t* test or the Mann–Whitney *U* test were used for the comparison of variables between the groups of controlled and uncontrolled asthma over the 12 months of follow-up. The Spearman rank correlation coefficient (R) was used to assess the relationship between sputum eosinophils and serum periostin levels, FeNO, and peripheral blood eosinophil count. The sensitivity, specificity, and predictive values of different thresholds of peripheral eosinophils (220 and 300 cells/µL) and FeNO (20, 40, and 50 ppb) for predicting sputum eosinophils > 3% were calculated. The level of agreement between the two allergologists in the identification of clinically relevant allergic asthma was evaluated with the kappa statistic.

Additionally, a logistic regression model with backward stepwise selection was used to assess factors associated with uncontrolled asthma at follow-up. Clinically relevant variables with a *p* < 0.2 in the bivariate analysis were included in the model as the independent variables, with uncontrolled asthma as the dependent variable. Moreover, the area under the ROC curve (AUC) for the regression model with the 95% confidence interval (CI) was calculated. Statistical significance was set at *p* < 0.05. The Statistical Package for the Social Sciences (SPSS) version 22.0 (IBM Corporate, Armonk, NY, USA) was used for the analysis of data.

Additionally, a comprehensive automated stratified exploratory data analysis process (AutoDiscovery, Butler Scientifics, Barcelona, Spain) was performed to identify hidden factors potentially associated with bad control of asthma. This process was carried out in each of the possible subgroups of the data set generated by means of a list of previously selected stratification factors. Subgroups or associations with a sample size less than 5, a sample size less than 1% of the total sample size, or a significance level α (two-sided test) equal to or greater than 0.05 were automatically rejected. Finally, an expert assessment of the registered results was carried out to select the most relevant results related to the original goals.

## 3. Results

### 3.1. Characteristics of Patients

The study population included 98 patients (26 men, 72 women) with a mean (SD) age of 47.2 (15.3) years (range 18–78). Adult-onset asthma was recorded in 61.2% of patients. Uncontrolled severe asthma was diagnosed in 86 patients (87.7%), with poor symptom control (ACT score < 20) in 56 (57.1%), obstructive pattern (FEV_1_ < 80% predicted) in 57 (58.1%), and more than one exacerbation in the previous year in 20 (20.4%). Main findings at the initial visit are shown in Table 1.

Allergic sensitization detected via the prick test was observed in 80 patients (81.7%), with polysensitization to allergens in 17 (17.3%). Sensitization to house dust mites (65.9%) and pet dander (43.9%) were the most frequent allergens. In 36 of these 80 patients (45%), two independent allergologists (kappa correlation coefficient 0.761) considered that they had clinically relevant allergic asthma. Details of allergic sensitization are shown in Table 2.

### 3.2. Sputum and Peripheral Blood Eosinophils

The mean percentage of sputum eosinophils was 4.7% (SD 6.3%) and the mean eosinophil count in the peripheral blood was 467 (median 400; interquartile range 300–545) cells/µL. Almost half of the patients (48.3%) had sputum eosinophilia (>3% eosinophils). The mean percentage of sputum neutrophils was 47.4% (SD 29.7%), and the percentage of patients with >61% sputum neutrophils was 41.7%. Based on sputum cellularity, 36 (36.7%) patients showed an eosinophilic profile, 12 (12.3%) a neutrophilic profile, 27 (27.6%) a mixed profile, and 23 (23.5%) a pauci-granulocytic profile.

**Figure 1:**
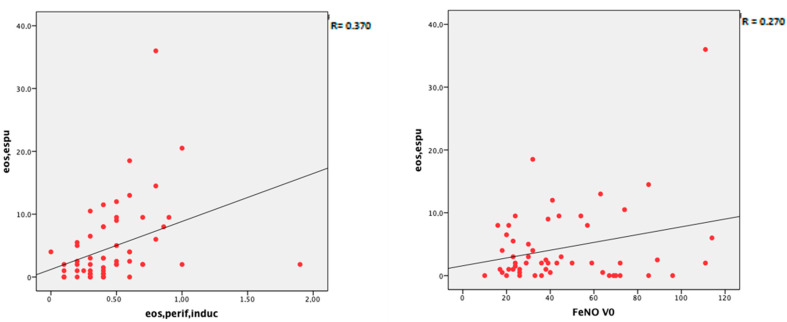
Correlation between sputum eosinophils and blood eosinophil count (**left**) and FeNO (**right**).

Sputum eosinophils showed a statistically significant correlation with FeNO values (R = 0.270, *p* = 0.04), the percentage of eosinophils in peripheral blood (R = 0.363, *p* = 0.004), and the eosinophil count in the peripheral blood (R = 0.370, *p* = 0.004) (Figure 1). Sputum eosinophils did not show a correlation with serum periostin (R = −0.078, *p* = 0.551).

The analysis of different thresholds of peripheral blood eosinophilia (200 and 300 cells/µL) and FeNO (20, 40 and 50 ppb) for predicting patients with >3% sputum eosinophils showed that an eosinophil count of >300 cells/µL was the cutpoint associated with the best sensitivity (72.4%), specificity (51.6%), and positive and negative predictive values (58% and 66%, respectively). FeNO > 20 ppb showed 92% sensitivity but 10% specificity and 46.9% and 60% positive and negative predictive values, respectively.

### 3.3. Inflammatory- and Immune-Related Parameters

The mean (SD) FeNO was 46 (33) ppb, and the mean serum periostin was 6947 (7801) pg/mL. The levels of cytokines in sputum samples are shown in Table 2. Inflammatory parameters of adaptive immunity were evaluated by measuring Th2, Th1, and Th17 populations and derived interleukins (Table 3). The innate immune pathway was also evaluated through quantification of ILC1, ILC2, and ILC3 (Table 2).

### 3.4. Asthma Control at Follow-Up

During the 12-month follow-up period, there were 48 patients in whom asthma was never controlled and 50 with controlled asthma, including persistently controlled asthma in 16 and occasionally controlled asthma in 34. Patients with uncontrolled asthma were older, showed a higher percentage of adult-onset asthma, presented more symptoms, and had poor quality of life and pulmonary function compared to controlled asthma patients (Table 4).

Differences in the level of asthma control according to the presence of allergen sensitization were not observed (77.1% in never-controlled asthma vs. 82% in controlled asthma, *p* = 0.627), but clinically significant sensitization (allergic asthma) was recorded in 26 (52%) patients with controlled asthma and in 15 (31.2%) with uncontrolled asthma (*p* = 0.037). Sputum eosinophils, blood eosinophil count, and the percentage of patients with sputum eosinophils > 3% were similar in the groups of uncontrolled and controlled asthma. The distribution of patients according to the profiles of sputum cellularity was also similar. There were no statistically significant differences between the groups of uncontrolled and controlled asthma in the levels of IgE, periostin, and FeNO.

The mean (SD) values of IL-4 in sputum samples were significantly higher in patients with controlled asthma (8.38 [7.4] pg/mL) than in those with uncontrolled asthma (3.1 (2.8) pg/mL) (*p* = 0.030). Differences in the inflammatory parameters of innate and adaptive immunity according to the level of asthma control were not found (Table S1, Supplementary Material).

**Figure 2:**
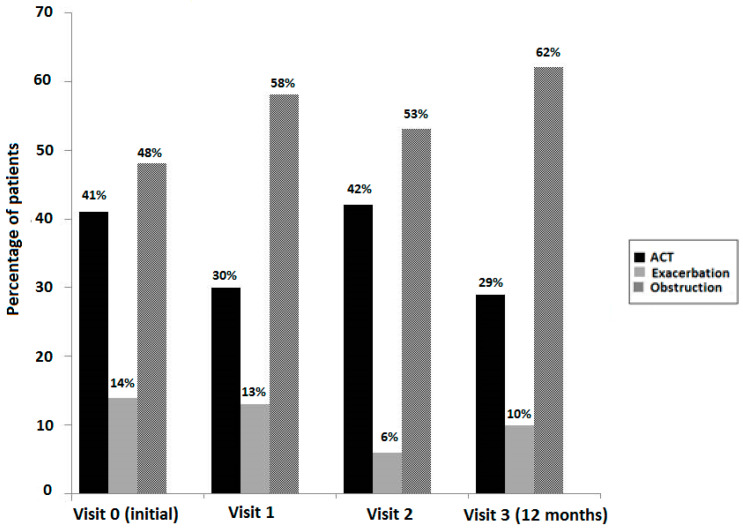
Percentage of patients with uncontrolled asthma at the initial visit and during the 12-month follow-up period considering the criteria of an ACT score < 20, number of exacerbations in the previous year, and airflow obstructive pattern (FEV_1_ < 80% predicted) separately.

**Figure 3:**
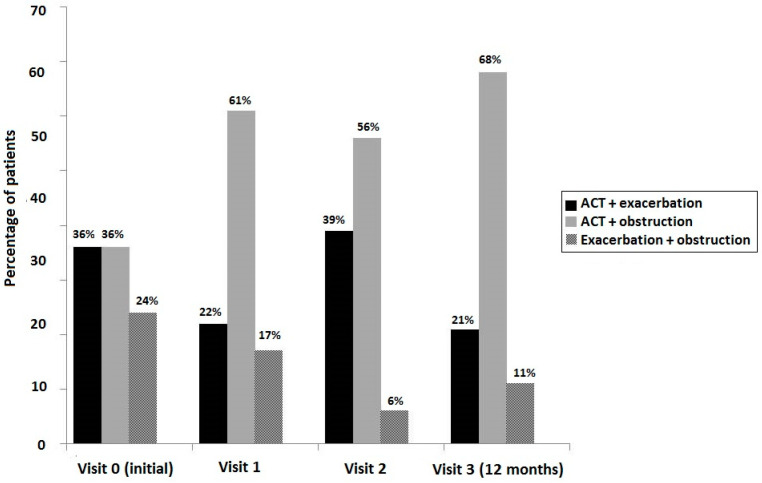
Percentage of patients with uncontrolled asthma at the initial visit and during the 12-month follow-up period considering the combined criteria of an ACT score < 20, number of exacerbations in the previous year, and airflow obstructive pattern (FEV1 < 80% predicted).

During the 12-month follow-up period, obstruction (FEV_1_ < 80% predicted) was the predominant finding in patients with uncontrolled asthma when criteria of ACT score < 20; lung function and exacerbations were either considered separately (Figure 2) or combined (Figure 3). According to this distinction, exacerbations showed a less important role in poor asthma control as compared to the combination of inadequate symptom control (ACT score < 20) and airflow obstruction.

In the multivariate analysis, the presence of an obstructive pattern (odds ratio (OR) 7.45, 95% CI 2.41–23.03, *p* < 0.0001) and the patient’s age (OR 1.045, 95% CI 1.005–1.086, *p* = 0.026) were the only two independent variables associated with uncontrolled asthma. The model showed an AUC of 0.76 (95% CI 0.66–0.86) (*p* < 0.0001).

**Figure 4:**
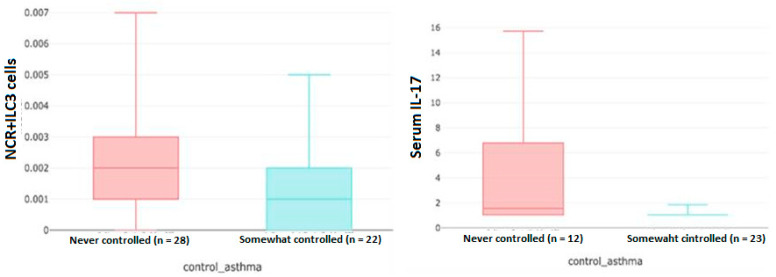
Boxplot of the relationship between NCR^+^ILC3 cells and asthma control at 12 months pf follow-up in patients diagnosed with asthma when they were older than 18 years of age (**left**). Relationship between serum IL-17 level and asthma control at 12 months of follow-up in the subgroup of patients with asthma duration between 20 and 35 years (**right**).

In the results of the automated stratified exploratory data analysis, it was found that serum IL-17 value was higher in patients with uncontrolled asthma at 12 months of follow-up in the subgroup of long-standing asthma (between 20 and 35 years). Additionally, the amount of NCR^+^ILC3 cells was higher in patients with uncontrolled asthma in the subgroup of patients in whom the diagnosis of asthma was established in adulthood (>18 years of age) (Figure 4).

## 4. Discussion

This study reports the characteristics of 98 adult patients with severe eosinophilic asthma and evaluates factors associated with uncontrolled asthma after a 12-month follow-up period. The present findings contribute to describe demographic and clinical features of these patients, their allergen sensitization pattern, the results of a large panel of biomarkers, and data of innate and adaptive immunity as well as Th1 and Th17 cell populations.

Most patients were women, with a mean age of 47 years, and presented with adult-onset asthma. At the baseline visit, there was a high rate of 87.7% of uncontrolled asthma, with airflow obstruction as the single cause of poorly controlled asthma in 48%. A 45% of the patients had a clinically relevant allergic sensitization associated. The prevalence of the different phenotypes among patients with severe uncontrolled asthma reported in the literature varies in the different clinical series of asthma patients according to criteria used for the definition of severity, level of control, and cutoffs of biomarkers. In a cohort study of medical record data of two databases of UK patients with active asthma, less than 1% of patients had severe uncontrolled eosinophilic asthma [5]. In patients seen at hospital units in Spain according to clinical criteria, a low prevalence of uncontrolled severe persistent asthma of 3.9% was found [4]. In a cross-sectional study of 179 patients with severe uncontrolled asthma carried out at 22 severe asthma units throughout Spain [21], general characteristics of patients were similar to those of our study, with a predominance of females in the fourth and fifth decades of life with adult-onset asthma. The present population, however, showed a lower BMI and higher sensitization to aeroallergens. The facts that peripheral eosinophilia was an inclusion criterion and that patient recruitment was performed in a multidisciplinary unit of severe asthma, where patients are also evaluated by allergologists, may explain the higher proportion of allergic asthma phenotype and the lower occurrence of obese-asthma phenotype. A similar number of patients received systemic corticosteroids (11.2% vs. 12.8%), but the use of biologics was clearly reduced in our patients since the study was conducted prior to the introduction of most biological therapies for Th2-high severe asthma.

At the time of the initial visit, therapy of asthma was optimized regarding doses of inhaled corticosteroids and LABA. It is surprising that exacerbations were not a risk factor for poorly controlled asthma. This could be attributed to the fact that all patients were recruited and visited every 3 months for severe asthma monographic consultations at a highly specialized multidisciplinary unit. It is probable that periodic medical and nursing control reduces the risk of exacerbation but does not have the same effect on other parameters of poor asthma control, such as airflow obstruction. In fact, prior exacerbations were only recorded as the main reason for poor asthma control at the initial visit in 14% of the patients, with persistence of symptoms and airflow obstruction as important determinants of poorly controlled asthma. In a report of French data from 129 patients included in a post hoc analysis of the international IDEAL study, asthma was poorly controlled in 67% of patients [22]. In the ARIETTA study, a prospective, longitudinal, international, multicenter real-world study of 465 patients, 57.6% had not suffered from ≥1 exacerbation in the previous year [23]. In 1186 patients included in the TENOR cohort, 44.5% of patients did not have histories of severe exacerbations [24].

Biomarkers for use in asthma associated with Th2 inflammation are still not well characterized [25]. The mean value of sputum eosinophils of 4.7% and the percentage of 48.3% of patients with eosinophilia > 3% may be lower than expected, which may be explained by treatment with high doses of inhaled corticosteroids and the fact that the sputum induction technique was performed when patients did not suffer from an exacerbation episode. Changing patterns of sputum cell counts during exacerbations of airways disease have been reported [26], as have high sputum eosinophil percentages after cessation of inhaled corticosteroids [27]. Moreover, a recent asthma exacerbation episode may cause a change in induced sputum inflammatory phenotype [28,29], particularly in patients with severe uncontrolled asthma [30].

Correlation of sputum eosinophils with other easily available biomarkers, such as peripheral blood eosinophils, FeNO, and serum periostin) [31,32] may be useful for asthma endotyping when the induced sputum technique is not available. In the present study, sputum eosinophils correlated with blood eosinophil count and FeNO values. However, the correlation coefficient between blood and sputum eosinophils of 0.363 is lower than the 0.59 reported by Wegener et al. [10] for a cohort of 110 patients with mild to moderate asthma. Based on our results, a peripheral eosinophil count > 300 cells/µL showed a sensitivity of 72% for predicting > 3% sputum eosinophils but with a specificity of only 52%. On the other hand, the correlation between FeNO and sputum eosinophils was low. In 75 uncontrolled asthmatic patients, Gao and Wu [33] reported a positive correlation (r = 0.4556) between sputum eosinophils ≥ 2.5% and a cutoff point of FeNO of 35.5 ppb. However, variability in the relationships between serum eosinophils and these biomarkers has been reported in other studies [26,34,35,36].

The analysis of IL showed low levels both in sputum and blood samples even using the CBA system, and although some studies have validated the use of some IL in blood and sputum [18], given the high cost of the analytical technique and the limited diagnostic reliability found in our study, the use of these parameters for endotyping or predicting control of severe eosinophilic asthma does not seem to be recommendable.

After 1 year of prospective follow-up, patients were grouped into those in which asthma was never controlled and those with controlled asthma at any time over the follow-up period. Interestingly the patient’s age and pulmonary function (FEV_1_) were variables independently associated with poor asthma control. In the TENOR II longitudinal study of patients with severe or difficult-to-treat asthma followed for 10 years, the lack of change of FEV_1_ might indicate that lung remodeling and irreversible lung disease had already occurred if these patients remained poorly controlled despite treatment [37]. In addition to persistently low FEV_1_, it has been shown that other factors, such as high short-acting beta agonist (SABA) use, previous severe exacerbation, incorrect inhaler technique, and especially poor adherence to treatment, had a significant influence on poor asthma outcomes [38]. The effect of poor inhaled technique or adherence to treatment appears to be minimized in our patients because they were attended in a severe asthma unit in which these two factors were specifically evaluated by specialized nurses. Persistent airflow obstruction with FEV_1_ < 80% predicted was the main factor associated with poor asthma control at the end of follow-up. However, it should be noted that in the present study population, biological therapies that target specific molecules and inflammatory pathways that have shown improvements of pulmonary function [39] were not used.

Other findings of the study were that higher levels of serum IL-17 and NCR^+^ILC3 cells were associated with poor asthma control at 1 year and onset of asthma in adulthood. Recently, it has been shown that IL-17 derived from Th17 cells and IL-8 and tumor necrosis factor-α derived mainly from macrophages are involved in the pathogenesis of steroid-resistant asthma and that these cytokines induce “NETosis”, a specific cell death of neutrophils, also involved in asthmatic airway inflammation [40]. When NETosis is induced in asthma, aggravation of inflammation and delay of tissue repair could occur, suggesting that NETosis may be associated with the development of steroid resistant asthma and persistence of airway remodelling [40]. Thus, in severe eosinophilic asthma, some subgroups may have poorer asthma control due to this phenomenon of the Th17 pathway characteristic of neutrophilic asthma. In contrast, high levels of IL-4 were related to better asthma control, probably in relation to the role of the IL-4 cytokine pathway in stimulating IgE inflammation.

Finally, in relation to allergy sensitization, 81.7% of patients were sensitized to at least one aeroallergen, a prevalence higher than the 50% described in most series of patients with asthma [4,21]. In our study, clinically relevant allergic asthma was considered in 45% of sensitized patients by two independent allergologists with high inter-rater reliability. The presence of allergic asthma was significantly more frequent among patients with controlled asthma than among those with uncontrolled asthma. However, differences between the groups of controlled and uncontrolled asthma in the profile of sensitization were not found. Although it is well recognized that atopic sensitization is an important risk factor for asthma both in children and adults, the potential role of allergy in severe asthma remains unclear [41]. In order to identify and differentiate phenotypes included under the umbrella of T2-high severe asthma (late-onset eosinophilic asthma, early-onset allergic asthma, and overlap phenotype), a complete allergic study should be performed and not only the qualitative assessment of allergy tests, which can lead to changes in the endotyping of this subgroup and consequently in therapeutic strategy.

Limitations of the study include the single-center characteristics of the study, the small sample size, and the fact that adult severe asthma is quite heterogeneous even if it has been pre-selected for the levels of eosinophils ≥ 220 cells/µL. However, these types of patients are those seen in real-world consultations and are different from patients enrolled in clinical trials with restrictive eligibility criteria. We used a clinical-practice-based criterion of peripheral eosinophilia (≥220 cells/µL) to select patients with severe eosinophilic asthma given that sputum eosinophilia is a not routinely available technique in all centers and is unfrequently used in routine daily conditions for the classification of these patients. The threshold level of blood eosinophils that categorizes a patient as having “eosinophilic asthma” varies greatly, and there is no standardized cutoff. We have chosen a lower cutoff value (220 cells/µL) compared to what is commonly used, taking into account that we were dealing with patients with severe asthma who had been chronically treated with medium-to-high doses of corticosteroids, which are known to reduce the baseline eosinophil count. Additionally, this cutoff value has shown a sensitivity of 86% and specificity of 79 in extrapolating central eosinophilia (sputum eosinophils ≥ 3%) in a replication cohort reported by Wagener et al. [10]. Additionally, evaluation of patients separated by asthma control and predominant sputum cellularity at baseline was not performed. Despite having optimized asthma treatment, most patients (87.7%) presented with uncontrolled asthma at the time of recruitment (controlled asthma was found in only 12 patients), and predominant sputum cellularity was not assessed. At follow-up, patients were grouped into those with uncontrolled (never controlled) and controlled (always/sometimes) asthma. However, the group of patients included in the category of controlled asthma is not homogeneous. Additionally, the effect of comorbidities was not evaluated. The study was carried out before the approval of advanced biological treatments, which could certainly have improved pulmonary function. On the other hand, the effect of treatment on changes of FeNO and eosinophil values during the follow-up period was not evaluated. Further studies may address this interesting aspect. In addition, assessment of risk factors for eosinophilic versus non-eosinophilic asthma would contribute to a better understanding of the reasons for poor asthma control.

## 5. Conclusions

In this study of patients with severe eosinophilic asthma in real-world clinical practice, 88% of them had uncontrolled asthma, 45% clinically significant allergic asthma, and 48% sputum eosinophilia at the initial visit. Sputum eosinophils correlated significantly with peripheral eosinophilia and FeNO. After 1 year, 49% of patients had uncontrolled asthma in all follow-up visits. The patient’s age and the presence of airway obstruction were independent factors associated with uncontrolled severe asthma. None of the inflammatory biomarkers evaluated here were found to be a robust predictor of poor asthma control. In patients with adult-onset and long-standing asthma, the IL-17 pathway may have a prognostic role with higher serum IL-17 levels associated with uncontrolled asthma, but further studies are warranted to confirm these preliminary findings.

## Figures and Tables

**Table 1:** Salient characteristics of 98 patients with eosinophilic asthma at the initial visit.

Variable	Patients n (%)	Mean (SD)
Female patients	72 (73.5)	
Age, years		47.2 (15.3)
Body mass index (BMI), kg/m^2^		26.5 (5.7)
Obesity, BMI > 30 kg/m^2^	20 (20.4)	
Duration of asthma, years		24 (14.3)
Age at asthma onset		
<11	30 (30.6)	
12 to 17	8 (8.2)	
≥18	60 (61.2)	
Family history of atopy	35 (35.7)	
Smoking status		
Never smoker	66 (67.3)	
Ex-smoker	23 (23.5)	
Current smoker	9 (9.2)	
Nasal polyposis	26 (26.5)	
Chronic rhinosinusitis	26 (26.5)	
Aspirin-exacerbated respiratory disease (AERD)	14 (14.3)	
Inhaled corticosteroids, µg budesonide equivalent		1405 (579)
Omalizumab treatment	15 (15.3)	
Systemic corticosteroids	11 (11.2)	
FEV_1_, L		2.2 (0.8)
FEV_1_, %		73.3 (20.5)
FVC L		3.21 (1.1)
FVC %		88 (26)
FEV1/FVC %		68 (14)
Eosinophil count the peripheral blood		467 (225)
ACT score		17.6 (5.5)
AQLQ score		5 (1.5)
Symptomatic patients (ACT score < 20)	56 (57.1)	
Obstructive asthma patients (FEV_1_ < 80% predicted)	57 (58.1)	
More than 1 exacerbation in the previous year	20 (20.4)	
Exacerbations		2.3 (3)
Severe uncontrolled eosinophilic asthma	86 (87.7)	

SD: standard deviation; FEV: forced expiratory volume in one second; ACT: Asthma Control Test; AQLQ: Asthma Quality of Life Questionnaire; FeNO: fractional exhaled nitric oxide.

**Table 2:** Details of allergens and sensitized patients.

Allergen	Number of Sensitized Patients	Specific IgE Mean (kU/L)
*Dermatophagoides pteronyssinus*	46	39.5
*Dermatophagoides farinae*	43	37.7
*Acarus siro*	14	7.8
*Lepidoglyphus destructor*	27	4.3
*Aspergillus*	9	5.4
*Alternaria*	6	0.7
*Penicillum*	1	2.9
Dog dander	30	18
Cat dander	28	8.8
Olive pollen	20	2.2
Plane tree pollen	9	4.5
Cypress pollen	5	1.3
Parietaria pollen	14	21.4
Mugwort pollen	7	8.4
Chenopodium pollen	6	0.1
Grass pollen	10	2.7
Cynodon pollen	5	2

**Table 3:** Results of inflammatory and immune variables in sputum and peripheral blood.

Variables	Mean (SD)
Fractional exhaled FeNO, ppb	46 (33)
Serum periostin, pg/mL	6947 (7801)
Sputum interleukins, pg/mL	
IL-4	6.63 (7.0)
IL-5	7.96 (13.3)
IL-8	4.36 (4.5)
IL-9	9.7 (9.19)
IL-13	5.59 (5.3)
IL-17F	17.28 (16.2)
Blood interleukins, pg/mL	
IL-4	15.8 (9.8)
IL-5	1.59 (5.0)
IL-8	15.8 (9.8)
IL-9	4.3 (4.5)
IL-13	1.77 (8.0)
IL-17	3.32 (9.3)
Cell populations	
Th1 effector, %	9.9 (6.2)
Th1 central memory, %	11.3 (4.9)
Th2 effector, %	3.2 (4.2)
Th2 central memory, %	9.1 (4.1)
Th17 effector, %	2.3 (1.2)
Th17 central memory, %	6.7 (4.0)
ILC1, ‰	0.12 (0.15)
ILC2, ‰	0.26 (0.32)
NCR^−^ILC3, ‰	0.15 (0.13)
NCR^+^ILC3, ‰	0.006 (0.016)

SD: standard deviation; IL: interleukin; NCR: natural cytotoxic receptor.

**Table 4:** Clinical characteristics of patients at 12 months of follow-up according to the level of asthma control.

Variable	Level of Asthma Control		*p* Value
	Never (*n* = 48)	Always/ Sometimes (*n* = 50)	
Female patients	35 (72.9)	34 (74)	0.903
Age, years, mean (SD)	51.1 (15.5)	43.5 (14.2)	**0.012**
Body mass index (BMI), kg/m^2^, mean (SD)	26.9 (6.3)	26.0 (4.9)	0.435
Obesity, BMI > 30 kg/m^2^	11 (22.9)	9 (18)	0.546
Duration of asthma, years, mean (SD)	25.3 (15)	22.7 (13.7)	0.376
Age at asthma onset			**0.029**
<11	12 (25)	18 (36)	
12 to 17	1 (2.1)	7 (14)	
≥18	35 (72.9)	25 (50)	
Family history of atopy	16 (33.3)	18 (36)	0.614
Never smoker	35 (72.9)	30 (62)	0.456
Chronic rhinosinusitis	11 (22.9)	14 (28)	0.582
Aspirin-exacerbated respiratory disease (AERD)	6 (12.5)	8 (16)	0.795
Some allergic sensitization	37 (77.1)	41 (82)	0.627
Some clinically significant sensitization	15 (31.2)	26 (52)	0.037
IgE levels, kU/L, mean (SD)	421 (581)	911.7 (2319)	0.161
FeNO, ppb, mean (SD)	51.2 (37.5)	41.2 (27)	0.161
Periostin, pg/mL, mean (SD)	6643 (7721)	7226 (7942)	0.720
Sputum eosinophils > 3%	27 (56.6)	21 (42)	0.311
Sputum eosinophils, %, mean (SD)	5.6 (7.8)	3.9 (4.6)	0.307
Blood eosinophil count, cells/µL, mean (SD)	402.2 (254)	418.6 (301)	0.776
Sputum cellularity profile			
Eosinophilic	14 (29.2)	18 (36)	0.582
Neutrophilic	5 (10.4)	8 (16)	0.647
Mixed	18 (37.5)	12 (24)	0.282
Pauci-granulocytic	11 (22.9)	12 (24)	0.854
Inhaled corticosteroids, µg budesonide equivalent, mean (SD)	1477 (565)	1366 (590)	0.230
Inhaled beta-adrenergic agonists	46 (95.8)	50 (100)	0.145
Inhaled anticholinergics	21 (43.7)	11 (22)	**0.026**
Omalizumab treatment	7 (14.6)	8 (16)	0.846
Systemic corticosteroids	7 (14.6)	4 (8)	0.302
Montelukast	32 (66.7)	35 (70)	0.723
FEV_1_, L, mean (SD)	1.81 (0.70)	2.56 (0.82)	**<0.001**
FEV_1_, %, mean (SD)	63.3 (19.1)	82.8 (17)	**<0.001**
ACT score, mean (SD)	16.4 (5.5)	18.8 (5.4)	**0.035**
AQLQ score, mean (SD)	4.64 (1.58)	5.35 (1.32)	**0.020**
Symptomatic patients (ACT score < 20)	33 (68.5)	23 (46)	**0.030**
Obstructive asthma patients (FEV_1_ < 80% predicted)	38 (79.2)	18 (36)	**<0.001**
More than 1 exacerbation in the previous year	11 (22.9)	9 (18)	0.546
Exacerbations, mean (SD)	2.15 (2.4)	2.44 (3.5)	0.630

SD: standard deviation; FeNO: fractional exhaled nitric oxide; FEV_1_: forced expiratory volume in one second; ACT: Asthma Control Test; AQLQ: Asthma Quality of Life Questionnaire. Data expressed as frequencies and percentages in parenthesis unless otherwise stated. Bold are those with statistically significant *p*-values (<0.05).

## Data Availability

Data of the study are available from the first author upon request.

## Supplementary Materials

The following supporting information can be downloaded at: https://www.mdpi.com/article/10.3390/jpm13071173/s1, Table S1: Innate and adaptive inflammatory parameters according to the level of asthma control during the 12-month follow-up.
