# Supplementary material for: Characterization and Factors Associated with Poor Asthma Control in Adults with Severe Eosinophilic Asthma
Source: J Pers Med. 2023 Jul 22;13(7):1173. doi: 10.3390/jpm13071173 (PMC10381894; doi:10.3390/jpm13071173)
Supplement: Supplementary file 1 [file jpm-13-01173-s001.zip › jpm-2482681-supplementary.pdf]

Table S1. Innate and adaptive inflammatory parameters according to the level of asthma control during the 12-month follow-up.

Variable	Level of asthma control		*P* value
	Never (n = 48)	Always/ sometimes (n = 50)	
Interleukins			
Serum levels, pg/mL, mean(SD)			
IL-4	3.07 (3.71)	2.29 (2.7)	0.249
IL-5	1.01 (1.9)	2.12 (6.7)	0.289
IL-8	16.01 (10.5)	15.6 (9.3)	0.860
IL-9	4.61 (4.6)	4.07 (6.7)	0.571
IL-13	2.45 (11.5)	1.14 (1.6)	0.433
IL-17	4.87 (13.1)	1.9 (2.6)	0.143
Sputum levels, pg/mL, mean (SD)			
IL-4	3.1 (2.8)	8.38 (7.4)	0.030
IL-5	4.97 (5.4)	13.7 (19.2)	0.586
IL-8	3.63 (2.29)	4.9 (6.6<9	0.388
IL-9	8.1 (7.1)	10.2 (12)	0.786
IL-13	4.7 (3.8)	6.01 (6.6)	0.914
IL-17	11.3 (0.3)	15.3 (16.4)	0.664
Cell populations, mean (SD)			
Th1 effector, %	9.78 (4.8)	10.13 (7.2)	0.810
Th1 central memory, %	10.9 (4.4)	11.7 (5.4)	0.505
Th2 effector, %	4.2 (5.9)	2.5 (1.6)	0.109
Th2 central memory, %	9.3 (3.7)	8.9 (4.5)	0.657
Th17 effector, %	1.4 (1.3)	2.18 (1.18)	0.416
Th17 central memory,%	7.3 (4.9)	6.26 (3.2)	0.291
ILC1, ‰	0.115 (0.15)	0.13 (0.15)	0.649
ILC2, ‰	0.26 (0.4)	0.27 (0.23)	0.918
NCR^{-}ILC3, ‰	0.143 (0.13)	0.153 (0.13)	0.493
NCR^{+}ILC3, ‰	0.007 (0.02)	0.0046 (0.01)	0.731
